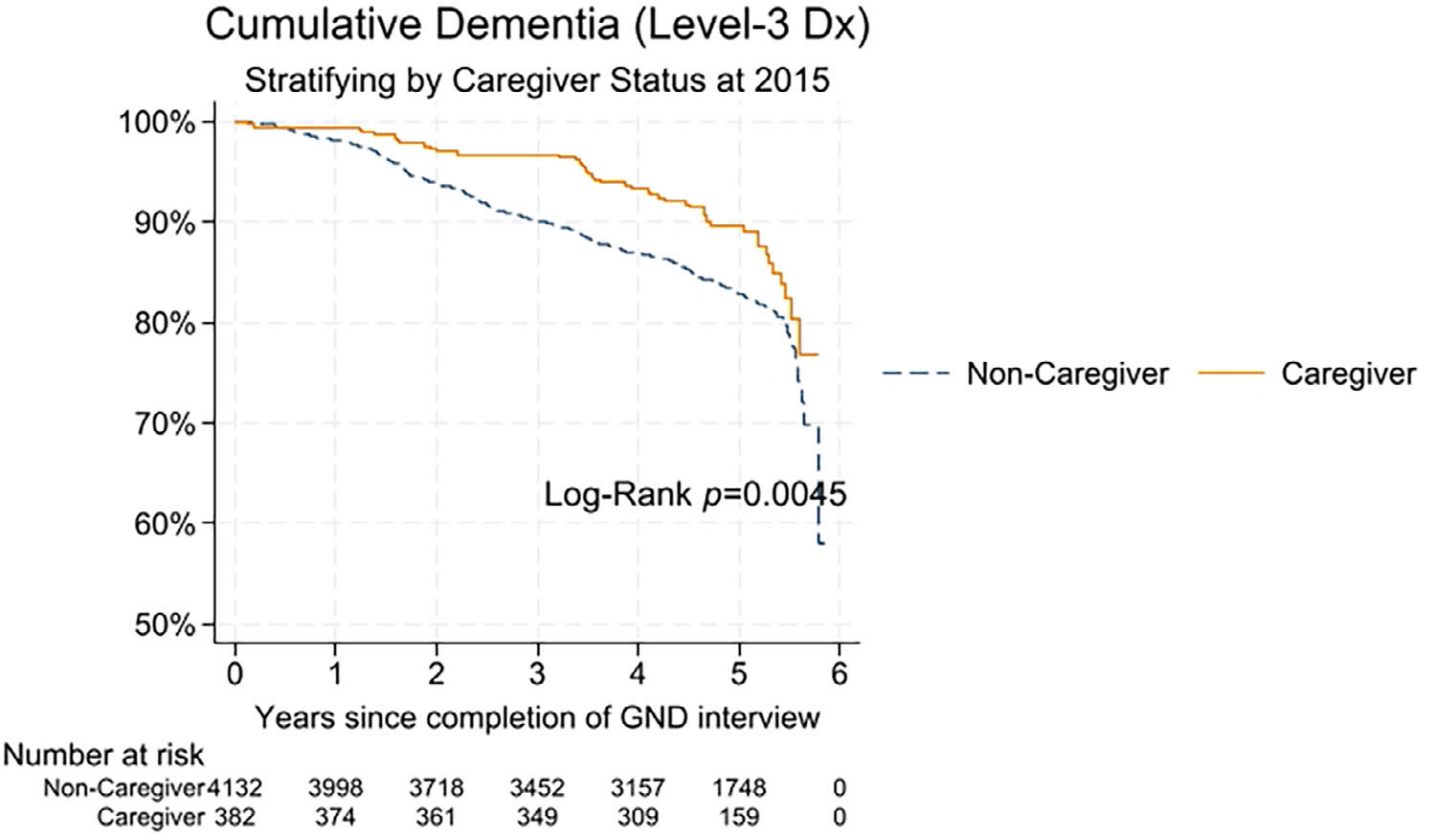# Caregiving and cognition: findings from the Atherosclerosis Risk in Communities (ARIC) Study

**DOI:** 10.1002/alz.084436

**Published:** 2025-01-09

**Authors:** Danielle Powell, Wuyang Zhang, Josef Coresh, Anna M. Kucharska‐Newton, Erin Kent, Martha Abshire Saylor, Shoshana Ballew, Katherine Ornstein, Jennifer A. Deal, Nicholas S. Reed

**Affiliations:** ^1^ University of Maryland, College Park, MD USA; ^2^ Johns Hopkins University, Baltimore, MD USA; ^3^ Johns Hopkins Bloomberg School of Public Health, Baltimore, MD USA; ^4^ University of North Carolina Gillings School of Global Public Health, Chapel Hill, NC USA; ^5^ University of North Carolina, Chapel Hill, NC USA; ^6^ New York University, New York, NY USA

## Abstract

**Background:**

Caregiving for older adults requires balancing multiple tasks ranging in complexity and demand. While often characterized as burdensome, there are also positive outcomes related to caregiving including potential benefits to health outcomes. Although older adults are themselves often caregivers, the association between caregiving and cognitive outcomes has not been routinely studied. We examine incident dementia among older self‐identified caregivers versus non‐caregivers. In secondary analysis, we examine the association between caregiving and 6‐year change in cognitive performance.

**Method:**

The Atherosclerosis Risk in Communities (ARIC) Study, an ongoing population‐based cohort study (since 1987) in four US communities, measured self‐reported caregiving status via telephone questionnaire in 2015. Cognitive measures (global, executive function, memory, language domains), adjudicated dementia status and participant characteristics were obtained during study visits. Accounting for sociodemographic characteristics, we examined the association between caregiving and development of incident dementia over a 6‐year period (2015 baseline to 2021) using Cox proportional hazard models and change in standardized global and domain‐specific cognitive factor scores over 6 years using linear mixed methods.

**Result:**

Among 4,514 ARIC participants (mean age 75.1, 60% female, 18% self‐reported Black), 382 self‐identified as a caregiver. No differences by caregiver status were observed in sociodemographic and health characteristics. Forty‐five (11.7%) caregivers experienced incident dementia over 6 years of follow‐up. In fully adjusted models, there was 30% decreased risk of incident dementia among caregivers (HR 0.69; 95% CI: 0.51,0.94) compared to non‐caregivers (Figure 1). Relative to non‐caregivers, caregivers had significantly better cognitive scores at study baseline. However, no difference in rate of change in global or domain‐specific cognition was observed.

**Conclusion:**

Caregiving was associated with decreased risk of incident dementia over 6‐years. We noted higher baseline cognitive performance but not rates of decline in caregivers versus non‐caregivers. Our longitudinal findings highlight potential positive health outcomes for older self‐identified caregivers. Further study of cognition among older adult caregivers would inform national and policy initiatives seeking to support caregivers’ health and function.